# Predictive chemistry: machine learning for reaction deployment, reaction development, and reaction discovery

**DOI:** 10.1039/d2sc05089g

**Published:** 2022-11-28

**Authors:** Zhengkai Tu, Thijs Stuyver, Connor W. Coley

**Affiliations:** a Department of Electrical Engineering and Computer Science, Massachusetts Institute of Technology 77 Massachusetts Avenue Cambridge MA 02139 USA ccoley@mit.edu; b Department of Chemical Engineering, Massachusetts Institute of Technology 77 Massachusetts Avenue Cambridge MA 02139 USA

## Abstract

The field of predictive chemistry relates to the development of models able to describe how molecules interact and react. It encompasses the long-standing task of computer-aided retrosynthesis, but is far more reaching and ambitious in its goals. In this review, we summarize several areas where predictive chemistry models hold the potential to accelerate the deployment, development, and discovery of organic reactions and advance synthetic chemistry.

## Abstract 2

This review outlines several organic chemistry tasks for which predictive machine learning models have been and can be applied.

## Graphical abstract

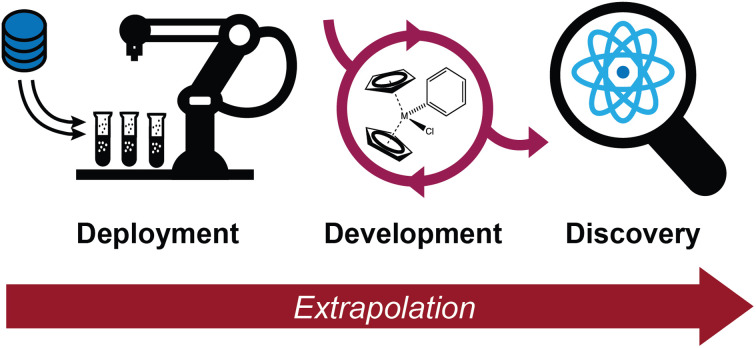

## Introduction

**Fig. 1:**
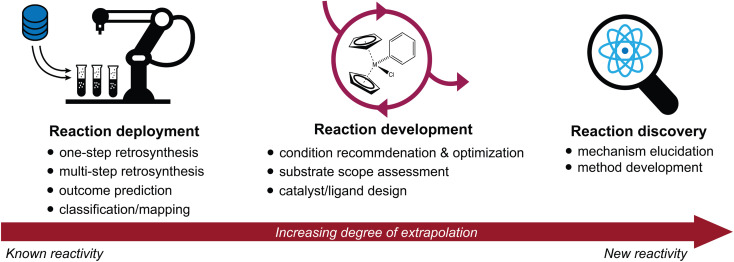
Overview of the three main categories of predictive chemistry tasks discussed throughout this review: reaction deployment, development, and discovery. It is useful to consider the extent to which each task represents an extrapolation from known reactivity to new reactivity.

Advances in the high-throughput generation and availability of chemical reaction data have spurred a rapidly growing interest in the intersection of machine learning and chemical synthesis.^1–4^ Deep learning approaches have achieved unprecedented accuracy and performance in a wide variety of predictive tasks; their potential to accelerate scientific discovery is therefore of immense interest.^5–7^ Here, we discuss recent advances in the application of machine learning to synthetic chemistry, divided in three categories (Fig. 1):

(1) *Reaction deployment*—learning from reaction corpora to identify trends and predict when known reactions apply to novel substrates or combinations thereof.

(2) *Reaction development*—accelerating the improvement or optimization of an existing chemical process, often in an iterative setting incorporating experimental feedback.

(3) *Reaction discovery*—creating new knowledge through the elucidation of reaction mechanisms or the discovery of unprecedented synthetic methods.

Progress in these areas has benefited from a “virtuous cycle” between chemistry and computer science experts, where the former identify pressing domain challenges and the latter design new computational tools to tackle them. As new algorithmic methods are developed, intended either for chemical problems or for the more widespread applications of image and language processing, the scope of synthetic problems able to be addressed by computational assistance expands. We encourage all synthetic and computational chemists to familiarize themselves with these applications and methods to identify (a) tools that can be directly incorporated into their R&D workflows, (b) additional applications where similar tools may be impactful, and (c) opportunities for developing novel algorithms.

This review will highlight progress towards building machine learning models that support synthetic chemistry in each of the areas of reaction deployment, development, and discovery. The progression through these three topics is meant to reflect an increasing degree of extrapolation from known reactivity to new reactivity. Throughout, we emphasize the major questions that models have been built to address, the myriad of approaches that have been developed to help address them, and some goals where further development is still needed. At times, we will go into some technical depth to describe and distinguish different models built for the same task, but these details may not be relevant for every reader.

## Preliminaries on machine learning and molecular representation

There are numerous reviews for machine learning in chemistry that provide an introduction to the field. Rather than explaining the basics of statistical learning, we instead redirect the reader to work by Strieth-Kalthoff *et al.*,^3^ Butler *et al.*,^8^ and Janet and Kulik.^9^ Here, we will only briefly mention a few key considerations in molecular representation and algorithm design.

Supervised learning problems are typically divided into regression and classification tasks, which seek to predict either a continuous scalar value or a discrete category. Both types of problems are ubiquitous in molecular machine learning and drug discovery applications (*e.g.*, in the form of quantitative structure–property relationship models), but cannot describe every task we discuss below. While the learning objective may be to predict reaction yield, rate, enantiomeric excess, *etc.*, some tasks require the prediction/generation of a *molecular structure*; for example, when predicting the product of a chemical reaction. Nevertheless, the types of tasks we will review are predominantly supervised learning problems wherein we try to recapitulate the relationship between input–output pairs derived from experiments or computational chemistry. When describing a supervised learning problem, it is essential to be precise about which factors should be considered part of the input, which factors are held constant, and which confounding factors are omitted due to missing data.

Molecular representation is perhaps the most fundamental aspect of molecular machine learning. In order for a model to learn the relationship between an input and an output, we must be able to describe the input in some objective, mathematical way. When working with reactions, we must choose how to represent the constituent molecules and other aspects of the reaction conditions. There has been a substantial amount of work on the former from cheminformatics and adjacent fields.^10^ The first consideration one makes is whether a molecular structure should be considered a rigid 3D object or a more flexible structure defined as a 4D conformer ensemble or a 2D/2.5D molecular graph. This choice is influenced by the learning problem, *i.e.*, whether the goal is to predict properties of an ensemble of 3D conformers, a specific 3D conformer, or the molecular identity. For most learning problems involving experimental reaction data, representing the molecular identity without restricting it to any individual conformation should be appropriate. However, computing properties of 3D conformers has proven to be an effective way to featurize catalysts and ligands for various learning problems, and 4D conformer ensemble inputs have been demonstrated to yield excellent results for, among others, solvation properties.^11,12^

Broadly speaking, molecular representations include *structure-based* fingerprints, SMILES strings,^13^ 2D graphs, and 3D conformations as well as *descriptor-based* vector representations using computed properties often inspired by physical organic chemistry. Descriptors may be directly derived from molecular structure and the two are by no means mutually exclusive.^14^ Each of these representations is compatible with a different set of machine learning model architectures (see Fig. 1 of Pattanaik and Coley^15^ for an illustration). What is considered “machine learning” is ambiguous; multivariate regression and PCA arguably count, but the implicit emphasis in this article will be on neural networks (*e.g.*, feedforward neural networks, graph neural networks (GNNs),^16^ the transformer^17^) and random forest (RF)^18^ models. Some components of reactions may be challenging to represent if they do not have a well-defined structure (*e.g.*, “air” as a reagent) or if they involve non-covalent bonds that are poorly described by SMILES strings or molecular graphs (*e.g.*, many organometallic complexes, including metallocenes). There is little standardization in, *i.e.*, no uniformly applied approach toward, how categorical reaction conditions are represented as inputs to machine learning models.

## Reaction deployment goals

**Fig. 2:**
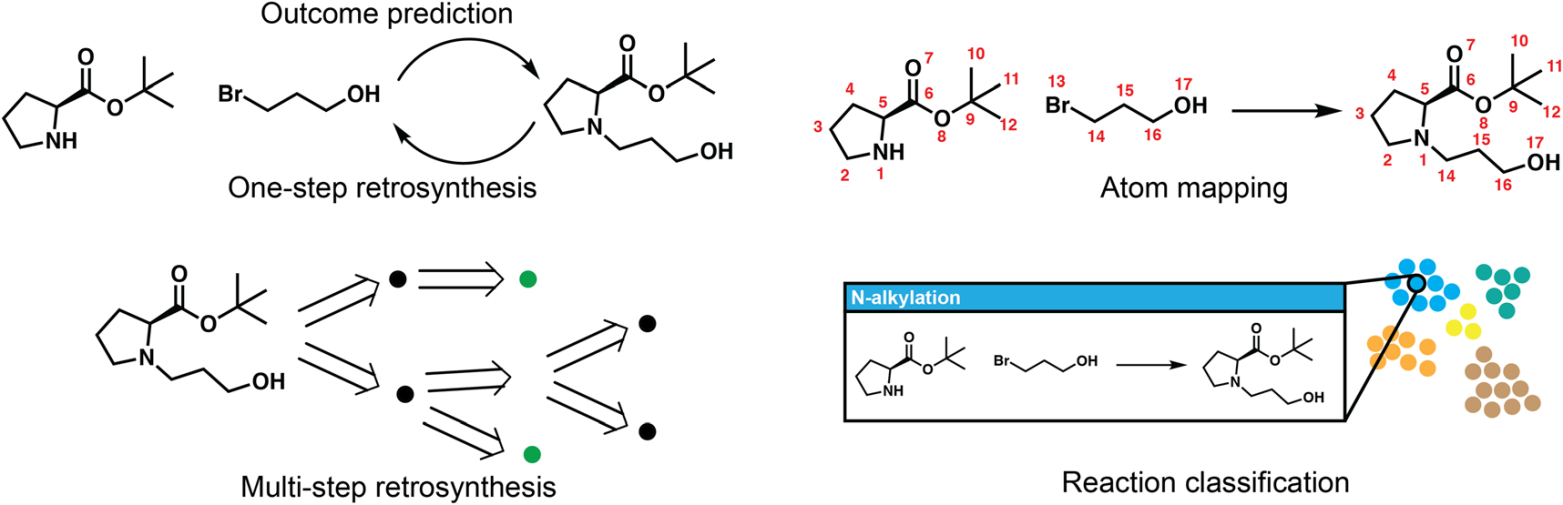
Overview of five key reaction deployment tasks. Reaction outcome prediction aims to predict the major product given the reactants. One-step retrosynthesis is the reverse task of proposing reaction precursors for new targets. The one-step models are called at each step of multi-step planning, which aims to propose synthesis routes that end in commercially/experimentally accessible building blocks. Atom mapping aligns the atoms on both sides of a reaction, and reaction classification maps reactions into distinct (human-interpretable) classes, both of which are complementary to the core synthesis planning workflow.

Reaction deployment involves the widespread task of retrosynthetic planning wherein new synthetic routes are proposed based on an algorithmic or statistical analysis of reaction data. These techniques do not aim to develop what a synthetic chemist would consider a “new reaction” (*i.e.*, a new method), but nevertheless may make predictions on new substrates *via* interpolation within reaction space. In addition to retrosynthetic planning, here we intend for it to also include the forward task of reaction outcome prediction, as well as other tasks to support information retrieval like classification and mapping (Fig. 2). Retrosynthesis and reaction prediction are both molecule-to-molecule transformations, but their approaches and evaluation diverge due to the one-to-many nature of retrosynthetic prediction and the lack of a single correct answer for model training and evaluation. Reaction prediction, generally simplified as major product prediction by recent works, is also arguably easier as we typically have all the heavy atoms in the reactant input, in contrast to retrosynthesis where atoms in the leaving groups have to be inferred.

### One-step retrosynthetic prediction

Models for one-step retrosynthesis aim to predict the “correct” reaction precursor(s) given the product molecule. Because there are many starting materials that could produce the target of interest, evaluation has focused on models' abilities to recapitulate experimentally-reported reactants within the highest ranked *k* options. The top-*k* accuracy (%) on the USPTO-50k dataset,^19^ a subset with approximately 50 thousand atom-mapped reactions mined from the US Patent and Trademark Office^20^ has emerged as a common (small) benchmark for comparison despite this underspecification; larger datasets of *ca.* 1 M from the USPTO have also been used (several versions of “USPTO-full”). Alternate metrics to top-*k* accuracy such as accuracy for the largest fragment^21^ and round-trip accuracy evaluated by a separate forward predictor^22^ have been proposed, and have since been used occasionally in parallel to top-*k* accuracy. The field has sometimes reported results when the reaction type-or class-is known and provided as part of the input, but this artificial setting has been decreasing in popularity. Some approaches have been evaluated on commercial (*e.g.*, Reaxys,^23^ CAS,^24^ Pistachio^25^) or in-house data (*e.g.*, electric laboratory notebook (ELN) data from AstraZeneca^26^ or Pfizer^27^), but results are also reproduced on USPTO-50k for most approaches.

Depending on whether these one-step models make use of *reaction templates*, which are reaction rules most commonly encoded using SMARTS patterns,^28^ they can be broadly categorized into template-based and template-free approaches; the latter can be further divided into graph-edit based and translation-based formulations.

#### Template-based approaches

Each template defines substructural patterns of reactants and products that codify, at least in a crude manner, a “rule of chemistry”. Reaction templates can be applied to product molecules to generate the corresponding reactants with the help of cheminformatics software such as RDKit.^29^ These templates can either be defined by expert chemists or algorithmically extracted from atom-mapped reactions,^23,30^ possibly using extraction tools such as RDChiral.^31^ Expert-defined templates have had use in retrosynthetic programs for decades and still form the knowledge bases of expert programs like Synthia;^32^ typically, in expert programs, templates are applied exhaustively and do not rely on models to downselect the most strategic templates.

The most basic data-driven template-based methods adopt a multi-way classification formulation to select the template that was extracted for the experimentally-recorded reaction given the product molecule structure. For example, NeuralSym^23^ uses extended connectivity (EC) fingerprints^33^ of product molecules as the input into a neural network which is trained to maximize the probability of the extracted template. Performance gains have been made possible with additional techniques like pretraining,^34^ refining template definition,^35–37^ clustering,^35^ or using additional features.^38^ Most notably, the state-of-the-art template-based method LocalRetro^36^ divides generic reaction templates into atom-change, bond-change and multiple-change templates, and trains three different classifiers accordingly.

Apart from the classification formulation, it is also possible to model one-step retrosynthesis as a retrieval or ranking problem. RetroSim^39^ retrieves the existing molecules that are most similar to given targets, and returns the associated templates as the results. MHNreact,^40^ on the other hand, encodes the template as well and trains a neural model to retrieve the most applicable templates for new molecules directly.

#### Template-free graph-edit based approaches

Despite attempts to refine template definition for retrosynthesis, there is always an intrinsic tradeoff between the generalizability and the specificity of templates. If the templates are defined too generally, they may not be able to capture sufficient information about chemical environments surrounding the reaction centers, and so the template may be used to propose disconnections that are not chemically feasible; if they are too specific, we may end up with an excessive number of templates each with few occurrences, making it harder to learn when its application would be synthetically strategic. Template-free approaches help mitigate this limitation. The first class of template-free methods are based on graph edits, modelling one-step retrosynthesis as a sequence of graph modifications that convert the target molecular graph into the reactant graphs. As most representative of such a formulation for retrosynthesis, MEGAN^41^ first determines a ground-truth order of actions (addition, deletion or modification of atoms and bonds) using some heuristic priority rules, after which a graph encoder-decoder is trained to predict the actions given the molecular graphs of the target or of the intermediates.

As variants of graph-edit based approaches, semi-template based methods that mimic the *synthon approach* to retrosynthesis have recently gained popularity. They first break the target into synthons (*i.e.*, hypothetical reaction intermediates), followed by a second stage to recover the reactants from predicted synthons. The reactant recovery process have been modelled as leaving group selection,^42^ graph generation^43^ and sequence generation^44,45^ conditioned on predicted synthons. In a similar way to template-based LocalRetro, G^2^Retro^46^ later refines the reaction centers to be bond-forming, bond-changing and atom-changing centers to enhance performance.

#### Template-free translation-based approaches

Graph-edit based approaches generally require atom-mapping to compute ground-truth graph edit(s), which complicates their application to large, potentially messy datasets (*e.g.*, ones missing some reagents or with ambiguous stoichiometry). This makes translation-based methods, the other category of template-free approaches, more attractive in certain scenarios. By modeling one-step retrosynthesis as a SMILES-to-SMILES machine translation problem, they normally do not need atom-mapping. The single-stage, end-to-end formulation also makes these models practically easier to train, even more so because they leverage sophisticated techniques from the domain of Natural Language Processing (NLP).

Translation-based baselines^47–51^ typically make use of sequence models including *Recurrent Neural Networks (RNN)* and the *Transformers*.^17^ The product SMILES string is first tokenized either character-by-character or with a *regex tokenizer*^52^ to, for example, keep four characters defining a chlorine atom “[Cl]” together as a single token. The sequence encoder learns to encode the tokens into some intermediate embeddings so that the decoder can autoregressively decode the reactant SMILES strings. Alternate molecular representations^53–55^ have been explored, and so have model architectures that use chemistry-relevant information of the target molecular graph.^24,56,57^ A number of translation-based approaches also directly borrow existing techniques from the NLP domain for performance improvement.^21,55,57–63^ Among the many performance engineering techniques is SMILES augmentation, which takes advantage of the fact that many different SMILES strings may describe the same molecular graph.^64,65^

#### Reranking, transfer learning and retrieval-based methods

Regardless of the one-step model used, the highest-ranked proposed precursors can always be corrected and/or reranked to yield better suggestions. Correction can be as simple as filtering out invalid SMILES,^21,24^ or with a separately trained neural syntax corrector to convert invalid SMILES into valid ones.^66^ As a more universal approach, Sun *et al.*^60^ and Lin *et al.*^67^ both train reranking models *via* contrastive learning, using the primary model predictions as *hard negatives* (decoys) that must be distinguish from the recorded ground-truth reactants.

There are also transfer learning approaches and retrieval-based approaches that are unfair to be compared with other approaches, but may nevertheless be relevant in some cases. For transfer learning, supervised pretraining with larger reaction databases may boost the model performance when transferred onto smaller datasets,^68,69^ although the performance gain when the model is given more reaction data is largely unsurprising. Similarly, some retrieval-based approaches to retrosynthesis make the prediction task easier by only retrieving from a predetermined set of molecules.^70,71^ This may, however, significantly limit the generalizability of the model since it assumes that a small collection of molecules includes every structure that could be used as a reactant.

### Multi-step retrosynthetic planning

Retrosynthetic planning for new targets of interest aims to propose full synthetic pathways, rather than merely the single-step transformations discussed so far. Single-step models can be applied recursively to the target product until we find the route(s) in which all building blocks are available (*e.g.*, present in some buyable database) or some termination criteria are satisfied (*e.g.*, maximal path length or search time). The extremely large search spaces of molecules and of reactions, however, render exhaustive search inefficient if at all possible. The number of candidate precursors to consider grows exponentially with increasing number of reaction steps as one proposes disconnection after disconnection. It is preferable and necessary to actively guide the search in the most promising directions.

**Fig. 3:**
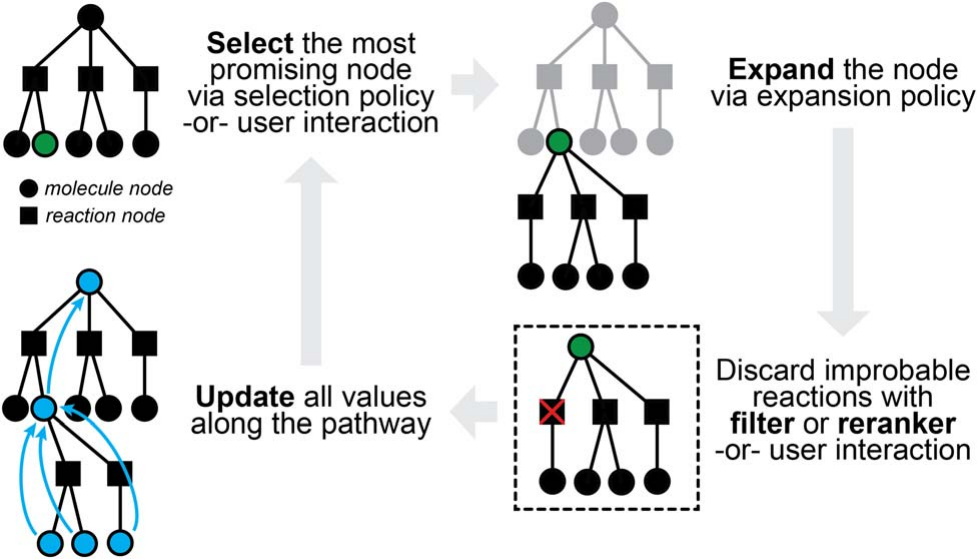
A sample iteration of multi-step planning, which takes a partially-expanded synthetic tree and chooses one chemical node to expand further.

The multi-step planning problem fits well into a general search framework with three phases, namely, selection, expansion, and update (Fig. 3). A synthesis pathway is first represented as a tree (or more generally a graph), with molecules and/or reactions being the nodes. In each search iteration, a *selection policy* is employed to find the most promising node(s) to expand (*i.e.*, the most promising molecule(s) to propose reactants for), which can either be based on heuristics or some *value function* of the node. This selection process is not too different from that in latest expert systems such as Synthia, which makes use of heuristically defined cost functions, possibly based on the structural complexity of a molecule.^32^ An *expansion policy* is then used to expand the selected node, for example, by applying a pretrained one-step retrosynthesis model. Relevant values along the path are then *updated* for use in future iterations. Multi-step planning has sometimes been viewed as a single-player or two-player game, which may have inspired the applications of *Monte Carlo Tree Search (MCTS)*^72^ and *Proof Number Search (PNS)*,^73^ both of which have been used for solving games in other contexts.

One fundamental challenge of multi-step planning is with the evaluation of proposed pathways. Assessing whether a synthetic route is “good” is highly subjective even for expert chemists.^74^ Unlike in one-step retrosynthesis where the top-*k* accuracy has been widely adopted as a standard metric (with known limitations), multi-step planning has few objective measures. Human evaluation with double-blind comparison between proposed and reported routes^75^ can be valuable, but is laborious and not scalable to large numbers of pathways. Some computable metrics that have been used include the success rate of finding viable pathways at different iteration limits, the average number of iterations for finding them, and the number of node visits, all on benchmark datasets again curated from USPTO (*e.g.*, on a test set of 190 target molecules^76^). While these metrics serve as basis for comparison, they are heavily oriented towards search efficiency rather than the *quality* and chemical feasibility of proposed routes. Various metrics have been proposed for quantifying route quality, including route length,^76,77^ average complexity of molecules in the route,^78^ and *tree edit distance (TED)*^79^ to a reference route.^80^ They are still far from perfect, and a consensus on evaluation has yet to be reached for the field. Because it is not possible to assess whether a proposed reaction would succeed with perfect accuracy (see later discussions of product prediction), we do not expect that compelling quantitative evaluations will arise in the foreseeable future.

#### Monte Carlo tree search (MCTS) for multi-step planning

As one of the most well-known approaches, Segler *et al.*^75^ were the first to combine a neural one-step model with MCTS. Every search step selects the best unexpanded node, expands the node with a template-based one-step model, and updates the scores along the synthesis pathway. The selection policy is formulated to achieve a balance between exploitation (*i.e.*, highest scoring nodes) and exploration (*i.e.*, unvisited nodes), with a variant of the *Upper Confidence bound applied to Trees (UCT)*^81^ used in AlphaGO.^82^ The selected node is then expanded with the one-step model, and only probable transformations are kept after filtering with a separately trained *in-scope filter*—a binary classification model meant to quickly check whether a reaction looks reasonable or not. As a distinct phase of MCTS, any new molecule generated during expansion will immediately be evaluated with a *rollout*, where a similar but more lightweight one-step model is iteratively applied to the new molecule. Depending on whether solutions (*i.e.*, pathways with buyable building blocks) are found, reward values will be assigned to the molecules, which are subsequently used for the update phase.

The MCTS approach and variants thereof have been implemented by ASKCOS^83^ and AiZynthFinder.^84^ Most notably, ASKCOS parallelizes the tree search in the original release, and augments the in-scope filter with a condition recommender and a forward predictor (discussed later); AiZynthFinder uses the same one-step model for expansion and rollout, trading efficiency during rollout for better quality of reward estimation.

#### Improvement of the search algorithm and structure

Other multi-step planning works can generally be viewed as replacing or improving various components under the general search framework. Some explore alternative search structures and/or algorithms such as AND-OR search and PNS, whereas others focus on improving the selection policy and rarely, the expansion policy.^85^ We will first review different search algorithms and/or structures, which are somewhat agnostic to the selection policy.

While the search tree can be easily modified to allow for node sharing (thereby turning it into a multi-tree as in ASKCOS or a hyper-graph as in Schwaller *et al.*^22^), quite a few recent works use AND-OR trees^76,77,85,86^ instead, whose early application to synthesis planning dates back to the pre deep-learning era.^87^ The AND-OR formulation enables alternative search algorithms to MCTS, such as *Proof Number Search (PNS)* to be used. We refer the reader to Heifets and Jurisica^87^ for details on how the proof/disproof numbers are defined for reactions (AND nodes) and molecules (OR nodes). Briefly, each reaction is represented as an AND node, whose state is true only if all of its successor nodes (which can only possibly be molecule nodes) are true. Each molecule is represented as an OR node, whose state is true if any of its successor nodes (which can only be reaction nodes) is true. The selection phase in PNS picks the OR node with the smallest proof number, or the AND node with the smallest disproof number. The expansion phase applies a one-step model similarly as in MCTS, and the update phase then updates proof and disproof numbers along the pathways, which in some cases may be generalized to depend on the value functions.^76^

Kishimoto *et al.*^77^ were the first to combine a template-based single-step model with PNS, which outperforms MCTS after incorporating heuristic scores based on reaction probabilities into the proof numbers of OR nodes. The performance was significantly improved later in Retro*,^76^ which reformulates the search as a single-player game by combining proof and disproof numbers into a redefined *reaction number* using an additional neural network value function estimator.

#### Improvement of the selection policy

The selection policy is a crucial component of the overall search, as it determines which precursors to pursue further. The UCT formula in MCTS can be easily modified, for example, by including a “dynamic c” parameter to dynamically force the exploration of nodes ranked low by the one-step model.^88^ Another common strategy is to better estimate the value function for any node without the expensive rollout. Injection of chemical heuristics in selection can be as simple as using a combination of reaction likelihood and complexity assessment score like *SCScore*,^89^ as was done in Schwaller *et al.*^22^ Similarly, ReTReK^90^ defines four heuristic scores to guide MCTS towards convergent synthesis, ring-forming reactions, and reactants with fewer reaction centers, harkening back to the early days of formalizing retrosynthesis where “x-oriented” (starting material-, stereochemistry-, topology-, *etc.*) strategies were proposed.^91,92^

While heuristic scores are generally cheap to compute, they do not take advantage of any data on known synthetic routes extracted from the literature. Retro*^76^ is among the first to utilize a learning-based value function estimator with a surrogate model. It starts by constructing routes for targets in the training set using existing reaction data in USPTO, after which the value (*i.e.*, the best entire route cost) for any target can be computed. A simple neural model is then trained to predict this value from structure, while maintaining preference for reactions within the routes over other reactions proposed by the one-step model. The ability of a model to navigate the search can be further refined with online learning, possibly in an iterative manner, with new training data generated from running the search.^88,93,94^ In this way, the model will get better at recognizing which intermediates are “most promising” and likely to connect back to buyable starting materials. Most recently, RetroGraph^95^ proposed to use a GNN on the search tree itself to parameterize the value function and learn which molecules to expand further, bringing its results to the state-of-the-art in terms of search efficiency on USPTO benchmarks with a few hundred test molecules.

#### Enumeration, ranking and clustering of pathways

The work we have reviewed so far mostly focus on improving the search efficiency, *i.e.*, increasing the success rate of finding a pathway with buyable building blocks while being faster and requiring fewer node visits. For practical use, however, it may be desirable to recommend more than a single viable pathway, which makes enumeration algorithms of *multiple* pathways relevant. CompRet^78^ ranks its enumerated pathways with heuristic scores that combine the longest path length, mean complexity (*i.e.*, mean SCScore^89^) of molecules in the route, and molecular similarities to reference routes. One can envision many different scoring metrics that can prioritize/deprioritize different proposals, such as ones estimating the cost of execution in a semi-automated lab.^96^ Ranking pathways by learned scores is also possible, for example, by training a tree-LSTM model to distinguish pathways with published reactions from artificial ones generated by a synthetic planner.^97^ Depending on the use case, pathways similar to patent-derived ones may either be preferred (*e.g.*, since they are safer to perform, arguably) or discouraged (*e.g.*, when patented routes are to be evaded^98^). While Mo *et al.*^97^ briefly experimented with clustering the routes based on their tree-LSTM embeddings and compares routes from the same or different clusters, Genheden *et al.* formally showed that some routes can be used as representatives of the cluster they are in (using a “tree edit distance”^79^ or a trained tree-LSTM model^99^), thereby reducing the total number of routes to be considered.

#### Retrosynthesis-derived models for synthetic complexity

To conclude the retrosynthetic planning section, we will briefly discuss a special use case of these planners as a filter during virtual screening. In the broader context of molecule or drug discovery, it is generally more preferable to fail early; we do not want to screen and/or optimize thousands or millions of molecules, only to discover that they are impractical to synthesize. Using retrosynthetic planners as filters are intuitively more advantageous than structure-based heuristic scores such as SAscore^100^ and SCScore,^89^ which may be inaccurate without considering any information of starting materials. However, running the pathway search for numerous compounds may be computationally prohibitive as each search can take a few minutes to run.

As one of the earliest attempts, RASA^101^ first implemented a retrosynthetic planner as formulated in Corey and Cheng^102^ with hundreds of transformations. They then regressed a linear model (on expert-labelled synthetic complexity scores for 100 medicinal compounds, using heuristic and route-derived features, some of which were also manually labelled), which can give correlation coefficients of as high as 0.8 when evaluated with unseen compounds. Several works fit route-derived scores in other ways, including expected path length,^103^ probability of successfully finding a viable path by a specific planner,^104^ or a pathway length score resulting from the retrosynthetic search itself.^105^ While these surrogate models speed up score computation by many folds and are agnostic to the choice of the planner, they are inherently limited by the planner from which the training data were generated.

### Reaction outcome prediction

Forward prediction is the task of predicting the product(s) of a reaction given reactants, and optionally, the conditions as well. The task is typically not fully specified in a quantitative way (*e.g.*, there is no consideration of reactant concentrations, among other aspects of the conditions), and is often simplified as predicting the single major product. In the context of reaction deployment, reaction outcome prediction mainly serves to check the plausibility of reactions proposed by the retrosynthetic planner, as well as to give an idea about patterns of selectivity and potential impurities or side products. While we focus our discussion on qualitative prediction tasks, it is worth noting that the broader scope of reaction outcome prediction may also include quantitative properties such as rate constants, yields, and equilibrium constants. These quantities are generally dependent on quantitative conditions, so they are used within reaction family-specific pipelines rather than general synthesis planning pipelines. We refer readers to Madzhidov *et al.*^106^ for a detailed review on quantitative prediction. Most notably, hybrid DFT/ML models have been developed to model the activation energies of nucleophilic aromatic substitution,^107,108^ one of the most well-studied reactions in organic synthesis.^109,110^

#### Template-based and template-free major product prediction

We can model forward prediction as reaction type classification^111^ or template classification,^23^ similar to the template-based approaches for one-step retrosynthesis. Given a set of reactants, the goal is to predict the type of reaction, which implicitly defines one or more products. A two-stage variant was later proposed by Coley *et al.*^112^ to predict the product molecules themselves, in which a pre-extracted set of around 1700 templates are exhaustively applied onto any reactants to generate a list of candidate products, which are then reranked by a learned reaction likelihood estimator to yield the final suggestions. In contrast to retrosynthesis, later developments for forward prediction have been dominated by template-free approaches: either graph-edit based or translation-based, with the only template-based competitor being LocalTransform^113^ which adapts a more general definition of reaction templates. Most notably, translation-based models such as the molecular transformer^114^ and follow-ups^21,24,55,63,115^ have shown clear advantages over the other methods on benchmark datasets such as USPTO_480k^116^ in terms of their accuracy in recapitulating experimentally-observed reaction products.

Graph-edit based approaches for reaction prediction were generally devised in a similar manner to those for retrosynthesis. Both two-stage pipelines^116–118^ and sequential graph-edit formulations^41,119^ are common. The two-stage formulations used for reaction prediction are similar to those for retrosynthesis, and actually predate them by multiple years. The major difference with retrosynthesis is that here the reaction centers are often atom pairs spanning multiple reactant molecules, rather than from a single target product. The sequential graph-edit formulation proposed in MEGAN,^41^ as we have discussed in the retrosynthesis section, works well for reaction outcome prediction too – by reversing the graph-edit sequence. An alternative to the sequential edit formulation is to consider it as a sequence of electron flow as in ELECTRO^120^ or a global redistribution of electrons as in NERF,^121^ where each step essentially predicts simultaneous graph edits (*e.g.*, bond breaking and bond forming), adding some chemical intuition to the models. Last but not least, the use of QM-augmented graph neural networks may serve as one form of chemical intuition, as the combination of structure-based and descriptor-based representations have achieved promising results on out-of-sample predictions in similar contexts.^122,123^

Adapting translation-based approaches for use in reaction prediction, on the other hand, is rather straightforward; it is still a SMILES-to-SMILES translation, except that now the inputs and the outputs are swapped. Indeed, the development of these approaches has almost followed the exact same trend as their counterpart for retrosynthesis, evolving from RNN-based sequence model^52,124^ into transformer-based molecular transformer,^114^ and then to the use of graph-aware encoders including GRAT^125^ and Graph2SMILES.^24^ Some of the model architectures and techniques discussed in the retrosynthesis section have also been applied directly to forward direction,^21,55,63,115^ confirming the effectiveness of techniques such as pretraining^63^ for forward prediction too.

#### Selectivity prediction for specific reaction types

Next to models targeting general (organic) reactivity, a variety of tools have been developed to target subtle reactivity questions for specific reaction classes. A major limitation that needs to be addressed when building a model for specific reactivity types is the relative scarcity of relevant training data. Several strategies have been explored to circumvent this issue. Pesciullesi *et al.*^126^ used transfer learning to build a data efficient transformer-based model capable of predicting regio- and stereoselective reactions on carbohydrates. Litsa *et al.*^127^ applied a similar approach to metabolic fate predictions, *i.e.*, prediction of drug metabolites. Zhang *et al.*^128^ in their turn combined transfer learning and data augmentation to train a transformer model on only a couple thousand of Baeyer–Villiger reactions. Tomberg *et al.*^129^ and Beker *et al.*^130^ made use of computed/physically-meaningful descriptors to improve the data efficiency/generalizability of their models, aimed at prediction of the regioselectivity of electrophilic aromatic substitution and Diels–Alder reactions, respectively. Finally, Struble *et al.*^131^ addressed the issue of limited data availability in their study of site selectivity in aromatic C–H functionalization reactions by designing their convolutional neural network as a multitask model, simultaneously learning across 123 types of functionalization with the goal of learning common patterns in the data between individual tasks.

### Reaction classification and mapping

Reaction classification and atom mapping are potential prerequisites for downstream use in machine learning, information retrieval when searching for similar reactions, the annotation of predictions, or the creation of labeled datasets for model training. In particular, atom mapped reactions are essential for many models for retrosynthesis and reaction prediction. They are required by template-based methods for template extraction, and by graph-based models to identify which subset of atoms are involved in the reaction, and which bonds are formed or lost. For these models, atom mapping is a crucial component of the data processing pipeline. Classification serves a less essential role in most workflows as its use is primarily in the analysis of historical trends in reaction popularity,^132^ the organization (clustering) and presentation of model predictions to users, or perhaps in evaluation to examine performance as a function of reaction type. Reaction classification also allows for type-conditioned prediction such as aforementioned selectivity prediction, as well as type-specific condition recommendation as will be discussed in the Reaction development section.

The predominant strategies for both involve the use of expert rules and heuristics. NameRxn exemplifies the expert strategy and is a widely used tool for reaction classification, naming, and atom mapping simultaneously.^133^ Each of several thousand reaction types is essentially defined by a reaction template (similar to those used for retrosynthesis, described above, even if not represented identically) in a 3-tier hierarchy; if a reaction template is able to recover the product when applied to the reactants and reagents, then the reaction type is assigned from the metadata of the template and the atom mapping is obtained from the newly generated product. NameRxn assignments are routinely used as ground truth labels for data-driven models, as discussed below.

Traditionally, atom mapping assignments have been obtained not through expert template application but through heuristic methods that pose the mapping process as an optimization.^134^ Many methods first find the minimum common substructure (MCS) between reactants and products, then identify the map that minimizes a graph edit distance^135^ subject to constraints about not changing atom types, penalties for breaking bonds that are not labile, et cetera.^136^ However, MCS alone may be insufficient for realistic reaction data that can require inferring stoichiometric ratios and missing reactant/reagent species. Jaworski *et al.*^137^ report a procedure to complement MCS with carefully-chosen expert rules, using a small collection of human-annotated reactions to demonstrate the comprehensiveness of their rule set. Comparison to some ground truth data is important given the lack of consensus across methods,^138^ despite the fact that there might be legitimate ambiguity in the “true” atom mapping due to mechanistic complexity. While there are relatively few data-driven approaches to atom mapping, a recent strategy of note is the extraction of attention weights in the transformer model for reaction prediction, a subset of which do seem to learn the principles of atom mapping.^139^ This is a logical yet clever use of the need for transformers to “remember” which atoms in the reactants have or have not been generated or copied to the products.

In contrast, there are many data-driven approaches to reaction classification given its direct connection to representation learning and the ease of formulating it as a supervised learning task: reaction → category. One benefit of ML-based classification/mapping algorithms is that they are more tolerant to “novel” chemistries; anecdotally, a large fraction of ELN reactions cannot be classified using rigid ontologies defined by reaction SMARTS. Assigning integer codes or identifiers to reactions has a long history in information retrieval (*i.e.*, by identifying reactions that undergo a similar structural transformation). But here, at some level, the goal is to contextualize a reaction in terms of human interpretable categories so there must be a manual component of defining these categories and labels. Schneider *et al.*^19^ use NameRxn assignments as the ground truth to train a classifier using a reaction fingerprint representation. This concept was later applied to a different reaction ontology, SHERC, still using reaction vectors from fingerprints of constituent components.^140^ Other representations of query reactions suffice, such as a continuous embedding learned from language models operating on SMILES strings that can be combined with a simple nearest neighbor model.^141^ Extensions of single-step classification include clustering of full synthetic routes as discussed above as a postprocessing step in retrosynthetic planning.

## Reaction development goals

**Fig. 4:**
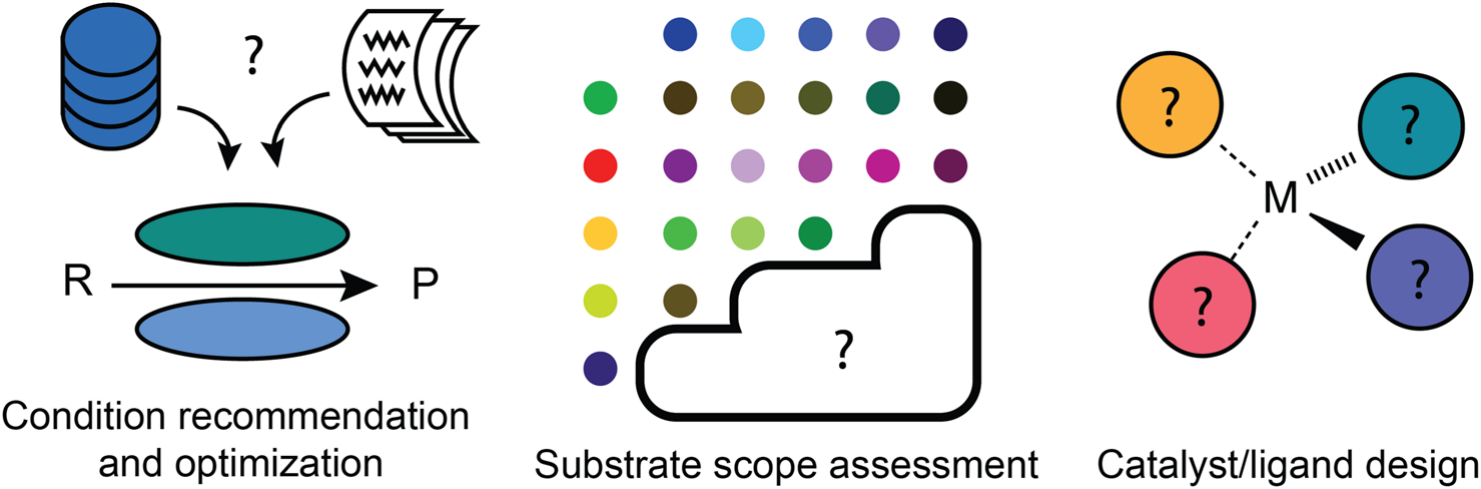
Overview of key reaction development tasks. Condition recommendation and optimization models can be built based on existing literature and electronic lab notebook data. Substrate scope assessment models have so far mainly been designed based on high-throughput experimentation results, where combinations of two or more reactant types are tested exhaustively. Catalyst/ligand design has been approached either through exhaustive screening campaigns, where ligand combinations are exhaustively enumerated from a library, or through generative modelling in recent years.

Reaction development has more to do with applying predictive models to accelerate the identification of a new and/or improved synthetic process (Fig. 4). It refines the general suggestion of what kind of transformation to use into a more actionable recommendation: what specific reaction conditions should be used? Does this type of reaction actually work for the substrate of interest? And if it does not seem to, what new catalyst or ligand combination might work? These questions do all affect the “deployment” of synthetic strategies, but require a greater level of precision and understanding of chemical nuance than most retrosynthetic and reaction prediction tools offer. For this reason, machine learning models may not be able to make a correct or complete prediction based on their training data and may instead be applied in an iterative workflow including experimental testing.

### Reaction condition recommendation and optimization

Relative to retrosynthetic planning, there has been little work done for the *a priori* prediction of reaction conditions. What has been done varies in terms of the level at which recommendations are made, *e.g.*, qualitative *vs.* quantitative, reaction family-level *vs.* substrate specific. It is easiest to envision an expert system making qualitative recommendations at the level of reaction families, as it is only necessary to recommend an example of “typical conditions” for that family. What is more useful in terms of actionability, however, is a substrate-specific recommendation that understands how the conditions should be tailored to the actual reactants to be used. A handful of data-driven models have been built for specific reaction types using previously acquired data from the literature or electronic lab notebooks, including solvent/catalyst classes for Michael additions^142^ and ligands for Pd-catalyzed C–N coupling.^143^ Once again, the quality of the training data is essential to build truly effective models. Beker *et al.*^144^ recently argued that in some cases, the level of noise and bias in literature data can impede the design of models that outperform literature popularity trends.

*Global* models, in contrast to these *local* (reaction family specific) models, are intended to predict suitable reaction conditions for “any” organic reaction of interest. Maser *et al.* demonstrated that a single model architecture based on a relational graph convolutional neural network could recover literature-reported conditions for Suzuki couplings, C–N couplings, Negishi couplings, and the Paal–Knorr reaction with an accuracy far exceeding a baseline approach that merely predicts the most popular conditions.^145^ This demonstration used data compiled from Reaxys that was further curated with more detailed reaction role assignments, *e.g.*, distinguishing categories such as metal, ligand, base, solvent, and additive. Just a few years prior, Gao *et al.*^146^ reported a broader model similarly based on the Reaxys dataset that, without filtering by reaction type, also showed a predictive accuracy significantly above the same popularity baseline. In principle, the domain of applicability of the model covers any hypothetical organic reaction that resembles a reaction type present in Reaxys.

One caveat is that all of these models discussed so far do not fully specify the reaction conditions; they omit details of concentrations, orders of addition, vessel setup, *etc.* and only specify the identity of the chemical species to use, primarily because this information is absent from their training data. There are at least two strategies to circumvent this limitation. The first is to curate or generate datasets where quantitative details are present, either for global models using richer data standards like the Open Reaction Database^147^ or for local models using focused experimentation where most aspects of the conditions are held constant.^148^ The second is to treat model predictions as initial guesses for subsequent optimization campaigns.

Empirical reaction condition optimization driven by algorithmic experimental design has existed for at least four decades.^149^ Briefly, model-based or model-free optimization techniques are used to propose reaction conditions in an iterative manner. One or more reactions are performed, the results are analyzed, and an algorithm proposes a new set of conditions to try next. While the problem formulation has not changed in years, recent trends include new treatments of discrete variables and a shift from statistical optimization methods, *e.g.*, using response surface models,^150^ to Bayesian Optimization (BO),^151,152^ with ML surrogate models^153^ or even deep reinforcement learning.^154^ Optimizing reaction yield with respect to continuous parameters like concentration, temperature, and time is the simplest setting as any number of continuous optimization algorithms (*e.g.*, BO, SNOBFIT) can facilitate experimental design; fortunately, this is perfectly complementary to the categorical reaction condition predictions that current data-driven models are able to make.

### Substrate scope assessment

A quintessential part of a synthetic methodology paper is the substrate scope table, which demonstrates the breadth of reactants with which the transformation is known to be compatible. This information is useful to chemists to understand when the transformation might be applicable to new substrates; it is similarly useful for computational algorithms, *e.g.*, retrosynthetic planners, to understand whether a proposed reaction step is likely to be successful. High-throughput experimentation can provide us with rich information about if (or quantitatively, how well) a reaction works for a given substrate. The role of machine learning in this setting can be to generalize to new substrates to predict their behavior *a priori*. The question of substrate scope is intimately related to reaction prediction, but in practice tries to be more quantitative in its prediction of yield/performance rather than merely providing a binary measure.

The retrospective analysis of HTE data and the use of non-random splits can probe a model's ability to generalize to new substrates. For example, Ahneman *et al.*^155^'s prediction of yields for C–N coupling reactions included an evaluation of generalization to unseen isoxazole additives. Unlike in a random split, the choice of molecular representation may have a large effect on performance. Simple one-hot representations of chemical species^156,157^ are inherently unable to generalize to new compounds. For this reason, testing “extrapolative” splits has become popular in these yield prediction tasks to gauge the value of different molecular or reaction representations.^158,159^ An important caveat of these studies is that data from HTE is qualitatively different from data that is typically published. In particular, a single paper might include only a dozen substrates; combining datasets from multiple papers describing the same reaction type will lead to confounding variables like the precise choice of conditions. That is, it can no longer be assumed that every aspect of the reaction is held constant besides the single substrate. When these confounding variables are present in a dataset, performance is unsurprisingly much worse.^160^ It is not fair to say that one setting is more or less realistic than the other, but the reality is that the majority of methods being developed for predicting reaction performance are validated on HTE data and cannot make use of the enormous diversity of reactions available throughout the literature.

**Fig. 5:**
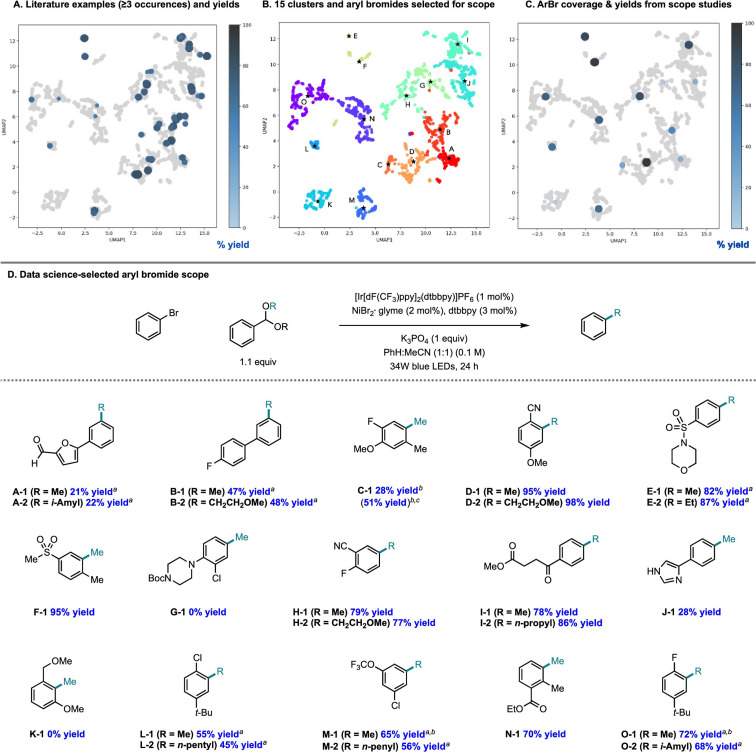
Systematic design of a substrate scope promoting diversity in descriptor space. Reproduced with permission from Kariofillis *et al.*^164^ Copyright 2022 American Chemical Society.

There is an additional use case for machine learning in substrate scope assessment that is prospective in nature. Rather than taking acquired data and trying to generalize to new substrates, surrogate models could be used to inform the selection of the most informative substrates to test: given a small number of known substrates and their yields, which new substrates/conditions should be tested in order to build the most accurate model? This is precisely an active learning formulation.^161^ Eyke *et al.*^162^ examined this question using existing HTE data by masking labeled data and allowing a model to choose which data points to unmask, demonstrating a significant improvement over random data acquisition (later simplified as a classification task by Viet Johansson *et al.*^163^); admittedly, more than just substrate identity are varied in these data. Kariofillis *et al.*^164^ describe a non-iterative approach tailored to substrate scope design wherein data science was used to inform the selection of reactants to test (Fig. 5). Starting from an initial pool of over 730 000 aryl bromides reported in Reaxys, those predicted to be compatible with Ni/photoredox catalysis were kept, featurized using 168 DFT descriptors, and clustered into 15 groupings from which the 15 centroids were selected for testing. Selecting these 15 molecules to be maximally diverse and representative of the overall chemical space of aryl bromides led to a wide distribution in performance, *likely* more varied than if 15 substrates had been hand-selected based on what an expert chemist assumed would succeed. We expect that a diversity-promoting method of selecting an initial screening set, followed by active learning where experiments are selected for maximal information gain, will gain traction as a systematic (and arguably less biased) approach to explore chemical reactivity.

### Catalyst/ligand design

Various excellent reviews have been written on the topic of computational design and optimization of (novel) catalysts and ligands in recent years.^165–169^ Hence, a detailed/exhaustive overview of this field will not be provided here. Instead, we will focus our discussion below on a selection of recent studies in which ML surrogate models (of varying complexity) have been used to predict and/or optimize the performance of novel catalysts. The supervised learning problem that is relevant for model-guided catalyst design resembles the ubiquitous quantitative structure–property relationship (QSPR) formulation where a molecular structure is mapped to a scalar property, and therefore benefits from extensive work in this area.

The least complex types of surrogate models are those based on multivariate regression and expert-curated descriptors. These models not only enable fast screening of extensive design spaces of potential catalysts, but can also facilitate insights in the underlying mechanism, through consideration of the respective correlation coefficients between individual descriptors and the selected target quantity. The best examples of this approach can be found among others in the work by Sigman and co-workers.^170,171^ Once a model is trained, hypothetical catalysts can be evaluated to downselect ones worthy of experimental validation.

Whenever non-linearity enters the picture, more advanced surrogate models are needed, and this inevitably comes at the expense of the aforementioned interpretability. For example, Denmark and co-workers used support-vector machines to anticipate the selectivity of chiral phosphoric acid-based catalysts and inform catalyst selection.^172,173^ Corminboeuf and co-workers have applied kernel ridge regression models to screen for suitable transition metal complexes for homogeneous catalysis, *e.g.*, for C–C cross-coupling^174^ and aryl ether cleavage reactions.^175^ Since computation of full reaction profiles for such multi-step reactions can be prohibitively expensive, a heuristic probe can help assess the suitability of screened complexes. Specifically, surrogate models predict the relative position of specific catalyst along a so-called “molecular volcano plot”: catalysts located close to the plateau of the volcano can be expected to exhibit ideal substrate–catalyst binding characteristics, and thus optimal thermodynamic/kinetic profiles.^176^ In its simplest form, ML surrogates can therefore help prioritize which calculations to run by recapitulating the results of first-principles simulations, as has also been extensively demonstrated and reviewed by Kulik and coworkers.^177^

Beyond surrogate models that enable the exhaustive screening of hypothetical catalyst/ligand structures, generative ML models have also been developed to propose new structures. Generative design itself is a decades-old technique,^178^ but deep generative design employing modern ML techniques has led to renewed interest. In one of the earliest examples of applying deep generative models to molecular design, Gómez-Bombarelli *et al.*^179^ proposed a variational autoencoder architecture in which discrete molecule representations are converted to and from multidimensional continuous representations. Within the latent vector space, gradient-based optimization can be performed, enabling a directed search for optimal functional compounds, without the need to evaluate/determine properties for the entire chemical design space (which, as the dimensions of the space grow can quickly become extremely time- and resource intensive). Generative molecular design has rapidly matured and now encompasses dozens of methods. An overview of more recent work on this topic can be found in the review by Elton *et al.*^180^

The utility of generative models for the design of novel catalysts has not necessarily been established, however. When candidate catalysts or ligands belong to combinatorial design spaces, genetic algorithms (GAs) provide an effective way to identify the most promising ones.^181^ Chu *et al.*,^182^ and more recently Laplaza *et al.*,^183^ have described the application of GAs to homogeneous catalyst optimization using computational models to assess performance. The use of GAs is in contrast to deep generative models that generate new structures atom-by-atom, fragment-by-fragment, SMILES token-by-token, *etc.*, which are arguably capable of making more “creative” ideas and exploring an even larger design space. The excitement around generative models (particularly in drug discovery applications, though the techniques translate well to catalyst and ligand design) should not overshadow the reality that generation or sampling is rarely the bottleneck in molecular discovery. We posit that the true bottleneck is evaluation, *i.e.*, having a good computational oracle function or an efficient experimental pipeline that lets one test the performance of new designs. Evaluation is commonly approximated by surrogate ML models as described above, but one cannot avoid the need for a well-defined evaluation protocol that ideally correlates with experimental performance.

To end this section, we want to highlight the importance of extensive datasets to accelerate these optimization tasks. In order to set up data-driven workflows to screen vast areas of chemical space for novel catalysts, vast libraries are needed to effectively exploit statistically derived structure–property relationships. Open-sourcing relevant datasets can facilitate – and democratize – the design and application of these workflows. Some catalyst/ligand datasets have been published in recent years such as Kraken,^184^ OSCAR,^185^ and the Open Catalyst Dataset,^186^ and we expect many more to be released in the near future.

## Reaction discovery goals

**Fig. 6:**
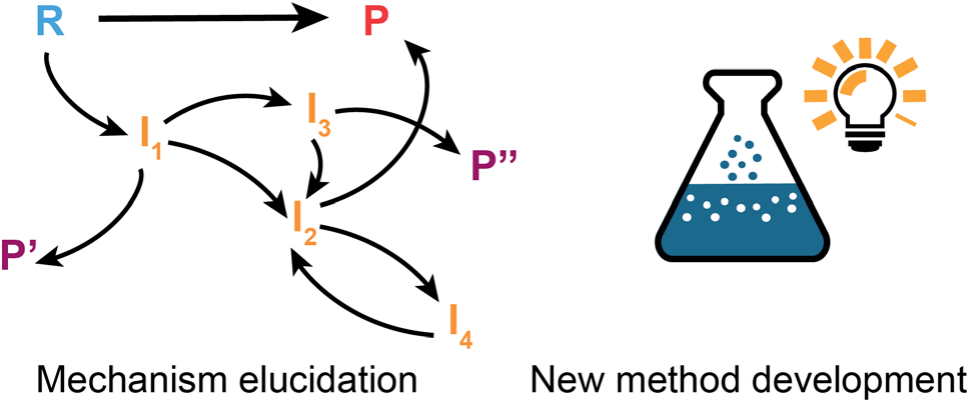
Overview of key reaction discovery tasks. Mechanism elucidation involves the explicit mapping of elementary reaction steps, and intermediates formed along the way, to achieve atomistic understanding of the chemical process under study. New method development involves the proposal of unprecedented reactivity by machine learning models that transcends trivial modifications of known templates.

Up to this point, the focus of this review has been on ML applications involving *known* chemistry, *i.e.*, interpolation based on existing data, which inherently implies that the prediction is constrained by precedents. It should be underscored however that machine learning approaches can also be employed to accelerate actual discovery of new chemistry. Under the term ‘*discovery*’, we understand here the creation of truly new knowledge, the invention of novel synthetic methods and/or the making of extrapolative leaps which transcend the current body of chemical knowledge.^5^ Before the advent of machine learning algorithms, such discoveries usually resulted from serendipity,^187^ or they were the result of (algorithm-based) exhaustive screening campaigns.^188^ Various aspects of algorithm/automation-accelerated chemical discovery have been reviewed as part of Gromski *et al.*^189^'s recent perspective. Here, we will limit ourselves to two challenging (sub)domains of chemical discovery which hold a lot of promise, yet have only received limited attention so far: ML-facilitated elucidation of unknown reaction mechanisms and novel method/reaction development (Fig. 6).

### Elucidation of unknown mechanisms

Most machine learning algorithms applied to chemical reactivity are mechanism agnostic, *i.e.*, they provide predicted outcomes given a set of inputs, but provide no information about *how* the chemical transformation actually transpires. The typical explanation of a reaction mechanism takes the form of an arrow pushing diagram and/or catalytic cycle. Nevertheless, it is sometimes possible to obtain mechanistic clues from a machine learning analysis indirectly. For example, in their study of Pd-catalyzed C–N cross-coupling reactions, Ahneman *et al.*^155^ identified a novel catalyst inhibition mechanism based on mechanistic clues obtained from a descriptor importance analysis within their constructed random forest models. In a similar vein, Sigman and co-workers have demonstrated on multiple occasions that mechanistic insight can be derived from descriptor based multivariate linear models.^190,191^ In certain cases, complex reactivity cliffs (analogous to activity cliffs in QSAR/QSPR) can be explained by simple univariate relationships, as in the case of a percent buried volume parameter for phosphine ligands.^192^ The distillation of predictive models into interpretable decision trees, even if the model itself is not inherently interpretable, can also provide insight as done by Raccuglia *et al.*,^193^ who derived a decision tree based on a support vector model (SVM) trained to predict the crystal formation of templated vanadium selenites. The resulting human-interpretable ‘*model of a model*’ was used to extract chemical hypotheses to guide future experimentation.

While these examples demonstrate that machine learning and the acquisition of mechanistic insights are not necessarily mutually exclusive, they can hardly be considered foolproof transferable strategies that can readily be deployed to any domain/application. After all, this type of approach implicitly requires the model featurization to have a direct connection to the ‘discovered’ mechanism, *i.e.*, there has to be a direct, human-interpretable connection between molecules' features and the phenomenon of interest. In the absence of prior knowledge (followed by careful feature engineering), this is not necessarily guaranteed and hence the success of these approaches at generating mechanistic understanding in part rests on serendipity (though the odds of success can be increased by casting a wide/diverse net of input descriptors/features of the model).

A more systematic approach toward the elucidation of *unknown* mechanisms may be an enumeration – followed by an evaluation – of all the different reaction pathways which might hypothetically connect reactants to products. Such a collection of many competing reaction pathways is generally denoted as a *reaction network*. Over the past decade, a wide range of computational codes have been developed for the analysis of such networks.^194^ One promising exploration strategy consists of reactive molecular dynamics (MD) simulations to sample accessible configurations according to a pre-defined thermodynamic ensemble, *cf.* the ‘*ab initio* nanoreactor’ developed by Martínez and co-workers.^195^ Limiting the appeal of this approach somewhat is the exuberant computational cost of this type of simulation – particularly when complex mechanisms involving many different compounds are analyzed – and the need for enhanced sampling techniques. It should be noted however that a lot of progress has recently been made on speeding up/reducing the computational demand of *ab initio* MD simulations with the help of machine learning, *e.g.*, through the development of neural network potentials^196–198^ and delta-learning approaches,^199^ though the extent of generalization of these techniques is not always clear, and extensive validation will be needed before these techniques can be applied in a true exploration mode.

**Fig. 7:**
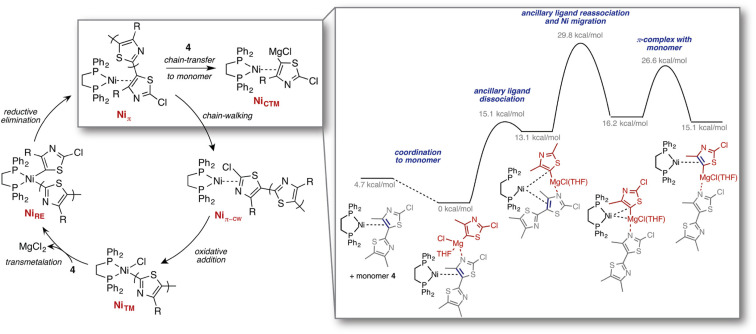
Computed mechanisms for the previously unknown chain-transfer to monomer pathway, competing with the regular chain-growth catalytic cycle, identified through reaction discovery computations. Reproduced with permission from Smith *et al.*^210^ Copyright 2016 American Chemical Society.

Other exploration approaches employ static quantum chemical calculations to estimate transition state structures and barrier heights associated with elementary reaction steps. For example, Maeda *et al.*^200,201^ explored Born–Oppenheimer PESs based on local curvature information, starting from an initial configuration. Graph-based rules originating from the concepts of bond order and valence have also been applied to identify such elementary reaction steps, *cf.* the work by Zimmerman on organic and organometallic reactions (Fig. 7),^202^ the reaction mechanism generator (RMG) code developed by Gao *et al.*^203^ for gas-phase (combustion) processes, and additional work on prebiotic reactions^204^ as well as by others.^205,206^ Finally, the CHEMOTON project by Reiher and co-workers represents a general, system-independent exploration approach based on heuristic rules derived directly from (static) electronic structure to explore complex reaction networks in an efficient and unbiased way.^207–209^

An inevitable issue that needs to be confronted during (complex) mechanism exploration is the combinatorial explosion of reaction possibilities: since the true mechanism of the reaction is unknown, pathways involving each and every combination of reactants/intermediates/products need to be probed in principle. As the number of identified stable compounds increases throughout the analysis, the systematic enumeration effort quickly becomes intractable. Machine learning offers a strategy to quell this combinatorial explosion by discriminating between combinations/pathways with respectively a high and low propensity to transpire. Provided enough training data, graph neural networks can both predict activation energies with almost chemical accuracy^211^ as well as propose viable transition state geometries,^212^ while Gaussian process based surrogate models have been used to elucidate heterogeneous catalysis mechanisms on the fly.^213^

Recently, several groups have started to employ reinforcement learning techniques to discover mechanisms in an automated and efficient manner.^214,215^ Instead of exhaustively screening all potential elementary reaction steps with a trained surrogate model, reinforcement learning involves an agent which is tasked with finding the most efficient pathway connecting reactants and products. Such pathways are constructed through the selection of sequences of actions, *i.e.*, elementary reaction steps, eliciting a varying ‘reward’ by the environment.^215^ By optimizing the received reward, the agent learns to select the most plausible reaction pathways on-the-fly. Reinforcement learning holds particular promise within the context of reaction network exploration since it bypasses the need to explicitly enumerate and evaluate all the combinations of elementary reaction steps and hence, it could be considered as the ultimate epitome of efficiency when it comes to reaction network exploration algorithms if sufficiently accurate. Its use does not mitigate the need to evaluate elementary reaction steps through first-principles or semi-empirical calculations.

Unfortunately, it is not realistic to avoid these calculations by directly training mechanistic predictors on experimental data (starting materials and final products), though the previously mentioned ELECTRO model—an autoregressive model that predicts reaction products by predicting linear electron paths—generates a “pseudo-mechanism” of sorts.^120^ Guided by electronegativity heuristics, ELECTRO generates arrow pushing diagrams that may describe certain polar reactions. The model is however incapable of understanding the role of catalysts or reagents which we may know to be essential for reactivity without additional supervision. Making use of expert annotations, *cf.* Baldi's ReactionPredictor,^216,217^ may be promising in this regard.

### New method development

In principle, conventional retrosynthetic and reaction prediction models are able to propose transformations that could be considered novel. In the simplest case, template-free retrosynthetic models can propose reactions that match a template not present in the training set.^58^ In practice however, the degree of extrapolation tends to be limited. “New” reactions proposed by reaction prediction models may involve trivial modifications of known templates with only slightly altered substrates. For example, Bort *et al.*'s work on GANs for the generation of Suzuki coupling reactions relies on filters to sift through many uninteresting reactions and flag those that exhibit novel reaction centers or unseen templates.^218^ Unambiguously novel mechanisms are exceedingly rare, and when they are in fact proposed by the model, the confidence by which these predictions are made is unclear. We have previously argued that the rate of false positives (mispredicted discoveries) is an important factor when trying to attribute a discovery to an algorithm or autonomous platform;^5^ reaction discovery is no different.

The lack of novelty exhibited by reaction prediction models developed so far is reasonable, as none of them were explicitly designed to *generate* novel reactions, though some first steps in this direction have been taken. For example, Segler and Waller^219^ model chemical reaction space as a graph, where molecules are represented by nodes and reactions by edges, and apply techniques from network analysis to predict new plausible links within the graph. Through more detailed analysis of the network edges that connect similar molecules, they were even able to suggest promising starting points for a high-throughput reaction discovery campaign. It should be noted here that the definition of a novel reaction as an unprecedented combination of known half reactions may not be agreeable to all chemists.

Recently, Su *et al.*^220^ considered the accuracy of the transformer model architecture on “zero-shot” reaction predictions. The goal of zero-shot learning consists of extracting accurate predictions for an unseen class of data points from a trained model, solely based on auxiliary information learned during training. With their experiments, Su *et al.* aimed to simulate the creative process behind the invention of the Chan–Lam coupling, which was inspired by the related Suzuki and Barton reaction classes. As such, the authors set up three different transformer models: one trained on the USPTO dataset without any Chan–Lam, Suzuki and Barton reactions, another one in which only the Chan–Lam reactions were removed from the USPTO dataset, and finally the USPTO dataset without Chan–Lam reactions but augmented with a set of additional Suzuki and Barton reactions. As one would expect, the first model performed poorly when evaluated on Chan–Lam reactions, reaching a top-1 accuracy below 5%, and the second model performed only moderately better (top-1 accuracy of almost 25%). With the fine-tuning of the additional Suzuki and Barton reactions however, the accuracy of the model shot up remarkably (top-1 accuracy > 55%), indicating that the transformer can indeed be made to extrapolate well from Suzuki and Barton reactions to the distinct, yet related Chan–Lam ones.

Despite this proof of concept that extrapolation to related reaction classes is possible in principle, it is unclear whether this approach can be applied in a more general/active manner due to the need to augment the training data with specific examples to reach a reasonable accuracy. Little is understood about *how* these models are generalizing, so little is known about what degree of extrapolation is reasonable to expect or what the rates of false positives or false negatives might be. There is still the issue, to reiterate, of how to generate hypotheses of new interesting reactions in the first place even if one has access to a “virtual flask” to anticipate the outcome; brute-force screening of reactant and condition combinations would at least be a baseline approach.

## Outlook

Many useful demonstrations of machine learning in predictive chemistry have emerged in recent years. Some tasks are well explored with many compelling solutions, such as retrosynthetic analysis, while others warrant new approaches and method development, such as mechanism elucidation. Throughout this review, we have focused on the progression of tasks from deployment, to development, to discovery, reflecting a scale of extrapolation ranging from “known” up to entirely “new” reactivity.

Despite their well-publicized successes, most machine learning tools are still not deployed routinely. Given the current level of interest in these techniques however, one can expect that they will become increasingly common and ubiquitous in modern chemical laboratories in the near future, especially as their performance is bound to continue improving as more relevant datasets and advanced algorithms become available. Already, retrosynthetic software is seeing increased adoption in industry whether using expert-defined transformations or data-driven programs as we have highlighted in this manuscript. In time, the mere idea of manually selecting reaction conditions for a Buchwald–Hartwig coupling or an amide bond formation reaction could very well be considered old-fashioned. A model that has learned substrate-optimal conditions from the collective work of thousands of experimentalists will be better equipped to choose a ligand than most synthetic chemists.

That being said, we should always keep in mind that the power of many neural models ultimately comes from their ability as universal function approximators, and is highly dependent on the data on which they are trained. A few recent studies have argued that a model may show overly optimistic performance if train and test sets are not split by scaffold^221^ or by source document,^24^ and that its predictive power may be limited by a lack of negative data points in literature.^222^ The black-box nature of many models renders interpretability challenging, and our confidence in neural models mainly relies on empirical verification (*e.g.*, by cross validation) with little theoretical guarantee. The ability to truly extrapolate is still the frontier. Few (if any) machine learning models have actually helped us to *discover* new insights and methods. Current models aren't designed to propose new, actionable information. A new generation of machine learning models should aim to operate at a more fundamental level, taking mechanistic considerations into account and/or being grounded in physics, so that more meaningful extrapolation may become possible. These models would complement descriptor importance strategies, where the bulk of the activity in machine learning assisted mechanism elucidation has been situated so far. We would like these models to yield new insights without being steered by human experts and eventually be capable of open-ended hypothesis generation and discovery. To work toward this goal, our own ongoing work in predictive chemistry is characterized by two transitions: from qualitative to quantitative, and from retrospective to prospective.

We would like to end this review by calling upon synthetic chemists and physical organic chemists to enter this burgeoning field of predictive chemistry. By defining new relevant tasks, as well as identifying failure modes of existing techniques, we can all help push predictive chemistry beyond the frontiers outlined above.

## Author contributions

ZT, TS, and CWC all contributed to writing the manuscript and the preparation of figures; ZT focused on retrosynthetic tasks; TS focused on forward synthetic and reaction discovery tasks; CWC focused on the remainder.

## Conflicts of interest

Authors declare no competing interests.

## Supplementary Material
